# Effect of CHK1 Inhibition on CPX-351 Cytotoxicity *in vitro* and *ex vivo*

**DOI:** 10.1038/s41598-019-40218-0

**Published:** 2019-03-05

**Authors:** Nicole D. Vincelette, Husheng Ding, Amelia M. Huehls, Karen S. Flatten, Rebecca L. Kelly, Mira A. Kohorst, Jonathan Webster, Allan D. Hess, Keith W. Pratz, Larry M. Karnitz, Scott H. Kaufmann

**Affiliations:** 1 Department of Molecular Pharmacology, Mayo Clinic, Rochester, MN USA; 2 Division of Oncology Research, Mayo Clinic, Rochester, MN USA; 3 Department of Pediatrics, Mayo Clinic, Rochester, MN USA; 4 Sidney Kimmel Cancer Center at Johns Hopkins, Baltimore, MD USA

## Abstract

CPX-351 is a liposomally encapsulated 5:1 molar ratio of cytarabine and daunorubicin that recently received regulatory approval for the treatment of therapy-related acute myeloid leukemia (AML) or AML with myelodysplasia-related changes based on improved overall survival compared to standard cytarabine/daunorubicin therapy. Checkpoint kinase 1 (CHK1), which is activated by DNA damage and replication stress, diminishes sensitivity to cytarabine and anthracyclines as single agents, suggesting that CHK1 inhibitors might increase the effectiveness of CPX-351. The present studies show that CPX-351 activates CHK1 as well as the S and G2/M cell cycle checkpoints. Conversely, CHK1 inhibition diminishes the cell cycle effects of CPX-351. Moreover, CHK1 knockdown or addition of a CHK1 inhibitor such as MK-8776, rabusertib or prexasertib enhances CPX-351-induced apoptosis in multiple *TP53*-null and *TP53*-wildtype AML cell lines. Likewise, CHK1 inhibition increases the antiproliferative effect of CPX-351 on primary AML specimens *ex vivo*, offering the possibility that CPX-351 may be well suited to combine with CHK1-targeted agents.

## Introduction

Despite advances in the molecular understanding of acute myeloid leukemia (AML), improvements in therapy are still needed. Most current regimens rely on treatment with the nucleoside analog cytarabine and an anthracycline for induction therapy. Depending on the subtype of AML, the driving oncogenic changes and the doses administered, these regimens have a 40–70% complete remission rate^1,2^. However, certain groups of patients, including those with antecedent hematological conditions or therapy-related AML, do particularly poorly with cytarabine/anthracycline-based induction therapy^3–5^.

A number of mechanisms of resistance to current AML induction therapy have recently been targeted, including increased expression of anthracycline exporters and increased activity of antiapoptotic BCL2 family members. However, resistance mechanisms involving the DNA damage and replication stress checkpoint pathways have received less attention. We and others previously reported that cytarabine activates the replication checkpoint kinases ATR and CHK1, which regulate a coordinated series of cellular responses that facilitate survival during replication stress^6–10^. Importantly, alterations that prevent activation of the replication checkpoint such as inhibition or downregulation of CHK1 sensitize AML cells to cytarabine^7–10^. CHK1 downregulation has also been reported to sensitize solid tumor cell lines to the anthracycline doxorubicin^11,12^, but this has not been tested using selective checkpoint kinase inhibitors in AML.

CPX-351 (Vyxeos^®^) is a liposomal formulation containing a synergistic 5:1 ratio of cytarabine and daunorubicin^13^. This formulation has a number of appealing properties, including the ability to kill AML cells harboring certain resistance mechanisms^14^ and a longer serum half-life than either cytarabine or daunorubicin^15^. CPX-351, which showed promising activity in AML clinical trials^16,17^, is now FDA approved for the treatment of therapy-related AML and AML with myelodysplasia-related changes^18^. Here we have assessed the impact of CHK1 inhibition on the cell cycle effects and cytotoxicity of CPX-351 in AML cell lines and clinical isolates.

## Results

### CPX-351 activates the replication checkpoint

**Figure 1:**
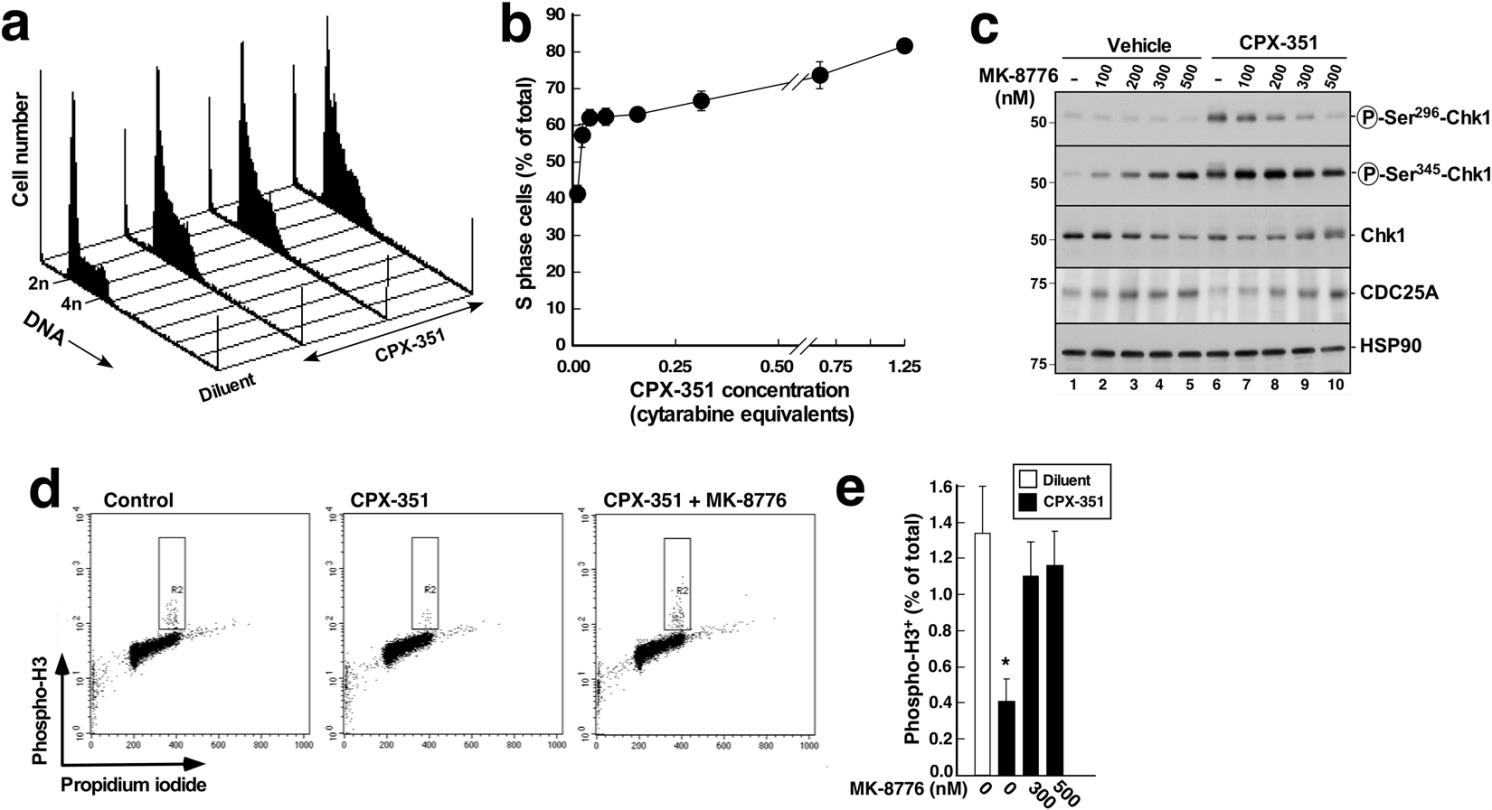
CPX-351 induces S phase arrest and activation of the ATR/CHK1 pathway. (**a**) DNA histograms of U937 cells treated for 24 h with diluent or CPX-351 (corresponding to cytarabine at 0.02, 0.04 and 0.08 µM in the fixed combination with doxorubicin) in the presence of the caspase inhibitor Q-VD-OPh^21^ (5 µM) to inhibit apoptosis-associated DNA degradation^19,20^. At the completion of the incubation, cells were stained with PI and analyzed by flow microfluorimetry as described in the Methods. (**b**) Percentage U937 cells in S phase after treatment with the concentrations of CPX-351 shown in panel a as well as additional concentrations. Results are mean ± sd of 3 independent experiments. (**c**) U937 cells were treated for 4.5 h with diluent (lanes 1–5) or CPX-351 (lanes 6–10) at a concentration equivalent to 2.5 µM cytarabine and 0.5 µM daunorubicin (abbreviated 2.5 µM cytarabine equivalents in subsequent figures) in combination with the indicated concentration of MK-8776 and immunoblotted for the indicated antigens. HSP90β served as a loading control. (**d**) Dot plots of U937 cells treated for 24 h with diluent or CPX-351 (corresponding to cytarabine at 0.31 µM in the fixed combination with doxorubicin) in the presence of 5 µM Q-VD-OPh and, where indicated, 500 nM MK-8776. At the completion of the incubation, cells were fixed, permeabilized, stained with PI and anti-phospho-Ser^10^-Histone H3 (Phospho-H3), and analyzed by flow microfluorimetry. R2 indicates the mitotic population. (**e**) Percentage U937 cells in M phase after treatment with diluent or CPX-351 along with MK-8776. Results are mean ± sd of 4 independent experiments. *Indicates p < 0.01 compared to diluent or treatment with CPX-351 in the presence of MK-8776.

In initial experiments, we examined the effects of CPX-351 on cell cycle distribution of the AML cell line U937. To assure that changes in propidium iodide staining were not a result of apoptosis-associated DNA fragmentation^19,20^, the caspase inhibitor Q-VD-OPh^21^ was included in these assays. Consistent with previous observations after cytarabine exposure^6,7,22^, U937 cells exposed to CPX-351 for 24 hours accumulated in S and G_2_ phases of the cell cycle, suggesting that the ATR/CHK1 replication checkpoint was activated (Fig. 1a,b).

Further experiments, performed in the absence of Q-VD-Ph, examined events at 4–8 hours after addition of CPX-351 to cells. Activation of the ATR/CHK1 pathway was confirmed by showing that CHK1 was phosphorylated on Ser^345^ (Fig. 1c, second panel), an ATR phosphorylation site required for CHK1 activation^23^, and on Ser^296^, an autophosphorylation site (Fig. 1c, top panel, lanes 1 and 6). Consistent with these findings, the CHK1 substrate KAP1 was phosphorylated on Ser^473^ and the ATR/ATM substrate H2AX was phosphorylated in Ser^139^ (Supplementary Fig. S1). In addition, the G2/M checkpoint^24^ was activated, as indicated by a decrease in the percentage of cells staining positive for the mitotic marker phospho-Ser^10^-Histone H3 after CPX-351 treatment (Fig. 1d,e).

In further experiments, the effects of the inhibitor MK-8776, which is highly selective for CHK1^25^, were examined. When combined with CPX-351, MK-8876 diminished CPX-351-induced CHK1 Ser^296^ autophosphorylation (Fig. 1c, top panel, lanes 6–10) and KAP 1 Ser^473^ phosphorylation (Supplementary Fig. S1) as well as increased the levels of CDC25A (Fig. 1c, fourth panel), a protein that is degraded in a CHK1-dependent manner to initiate the replication checkpoint^26^. Moreover, MK-8776 partially restored the percentage of cells that stained for phospho-Ser^10^-Histone H3 (Fig. 1d,e), suggesting that the G2/M checkpoint has also been blunted^24^.

### Effect of CHK1 inhibition or downregulation on CPX-351-induced apoptosis

**Figure 2:**
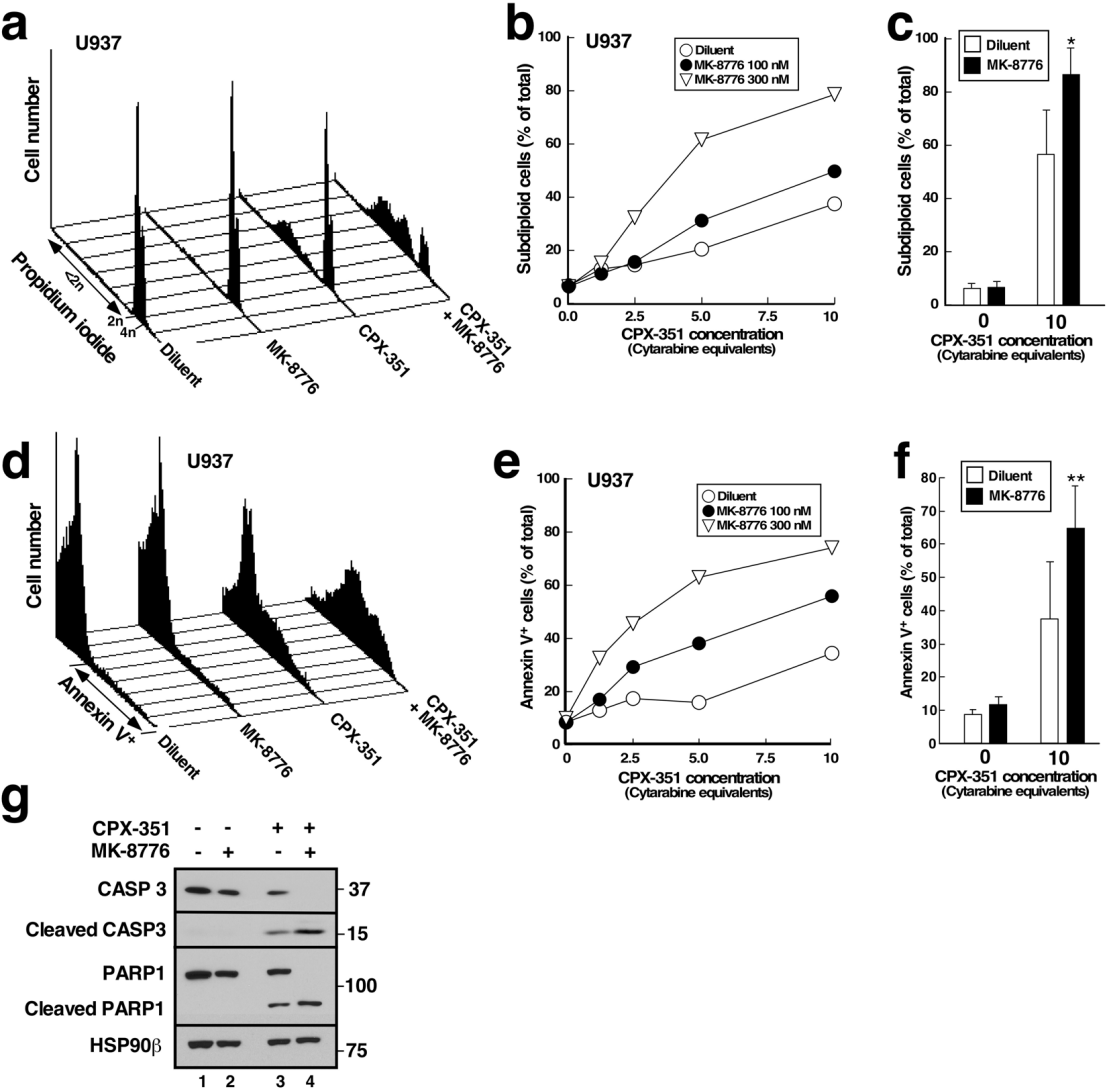
CHK1 inhibitors enhance CPX-351-induced apoptosis in U937 cells. (**a**–**c**) U937 cells were treated for 24 h with CPX-351 in the absence and presence of MK-8776, stained with PI and subjected to flow microfluorimetry. (**a**) DNA histograms from samples treated with diluent, 300 nM MK-8776, CPX-351 corresponding to 10 µM cytarabine, or the combination. (**b**) Summary of subdiploid DNA content in U937 cells shown in panel a and additional samples in the same experiment. (**c**) Summary of 10 independent experiments under conditions shown in panel a. Error bar, ±sd. *p < 0.0001 relative to CPX-351 plus diluent. (**d**–**f**) U937 cells were treated for 24 h with CPX-351 in the absence and presence of MK-8776, stained with Annexin V and analyzed by flow cytometry. (**d**) Histograms from samples treated with diluent, 300 nM MK-8776, CPX-351 corresponding to 10 µM cytarabine, or the combination. (**e**) Summary of annexin V binding in U937 cells shown in panel d and additional samples in the same experiment. (**f**) Summary of six independent experiments under conditions shown in panel d. Error bar, ± sd. **p = 0.011 relative to CPX-351 plus diluent. (**g**) After U937 cells were treated for 24 h with diluent, 300 nM MK-8776, CPX-351 at 5 µM cytarabine equivalents, or the combination, whole cell lysates were subjected to SDS-PAGE and blotted for procaspase-3, cleaved caspase 3, or PARP1. HSP90β served as a loading control.

In a parallel set of experiments, we omitted Q-VD-OPh and examined the ability of CPX-351 to kill AML cell lines by assessing DNA cleavage (Fig. 2a–c), phosphatidylserine externalization (Fig. 2d–f), PARP1 cleavage (Fig. 2g) and nuclear fragmentation (Supplementary Fig. S2), four separate hallmarks of apoptosis. For these studies, CPX-351 concentrations corresponding to those achieved in AML patients at the FDA-approved dose (equivalent to 5–10 µM cytarabine over the first 24 h)^15^ were applied. Each of these assays demonstrated that CPX-351 induced U937 cell apoptosis, which was increased by MK-8776 in a dose-dependent manner. In particular, MK-8776 increased the amount of CPX-351-induced DNA cleavage (Fig. 2a–c), phosphatidylserine externalization (Fig. 2d–f), nuclear fragmentation (Fig. S2) and caspase-mediated proteolysis of PARP1 (Fig. 2g). Additional analyses showed that MK-8776 enhanced the action of cytarabine and daunorubicin individually (Supplementary Fig. S3), demonstrating that MK-8776 impacts the cytotoxicity of both active agents in CPX-351.

**Figure 3:**
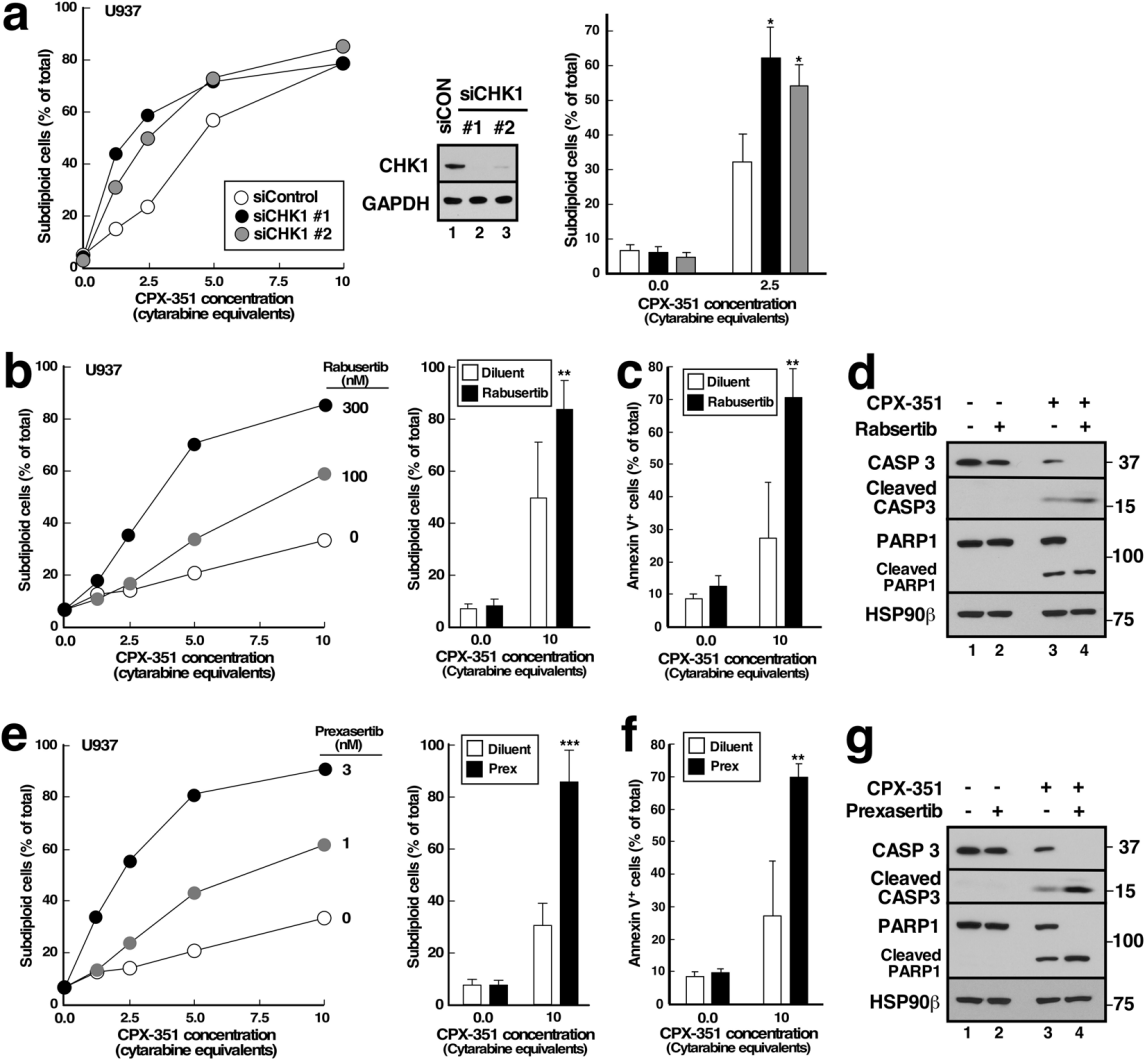
CHK1 siRNA and additional CHK1 inhibitors enhance CPX-351-induced apoptosis. (**a**) U937 cells were transfected with two different CHK1 siRNAs (siCHK1) or control luciferase siRNA (siLuc), incubated for 24 h, exposed to the indicated concentrations of CPX-351 for 24 h, stained with PI, and subjected to flow microfluorimetry. Left panel, results of one experiment. Middle panel, immunoblot of cell lysates prepared from cells 48 h after transfection with control or CHK1 siRNAs. Right panel, summarized results of 3 independent experiments. *p < 0.01 relative to control siRNA samples treated with CPX-351. (**b**–**g**) U937 cells were treated for 24 h with diluent or CPX-351 in the absence of presence of the indicated concentrations of rabusertib (**b**) or prexasertib (**e**) or in 300 nM rabusertib (**c**,**d**) or 3 nM prexasertib **(f**,**g**) in the absence or presence of CPX-351 at 10 µM cytarabine equivalents (**b**,**c**,**e**,**f**) or 5 µM cytarabine equivalents (**d**,**g**) and examined for subdiploid DNA by flow microfluorimetry (**b**,**e**), annexin V binding (**c**,**f**) or cleavage of procaspase-3 and PARP1 (**d**,**g**). In b and e, left hand panels show dose-response curves from individual experiments. Bar graphs show mean ± sd from 6 (**b**), 4 (**c**,**f**), or 3 (**e**) independent experiments. ** and ***p < 0.02 and p = 0.003 relative to CPX-351 plus diluent.

In further experiments, siRNA-mediated CHK1 downregulation also enhanced CPX-351-induced apoptosis (Fig. 3a), confirming that loss of CHK1 signaling rather than an off-target effect of the kinase inhibitor was responsible for the sensitization. Moreover, additional inhibitors with selectivity for CHK1, including rabusertib^27^ and prexasertib^28^, also enhanced the cytotoxicity of CPX-351 (Fig. 3b–g), with similar effects demonstrated by analysis of DNA cleavage (Fig. 3b,e), annexin V binding (Fig. 3c,f), or caspase-mediated proteolysis (Fig. 3d,g). These observations argue that enhancement of CPX-351-induced apoptosis is not unique to one CHK1 inhibitor or one assay methodology.

**Figure 4:**
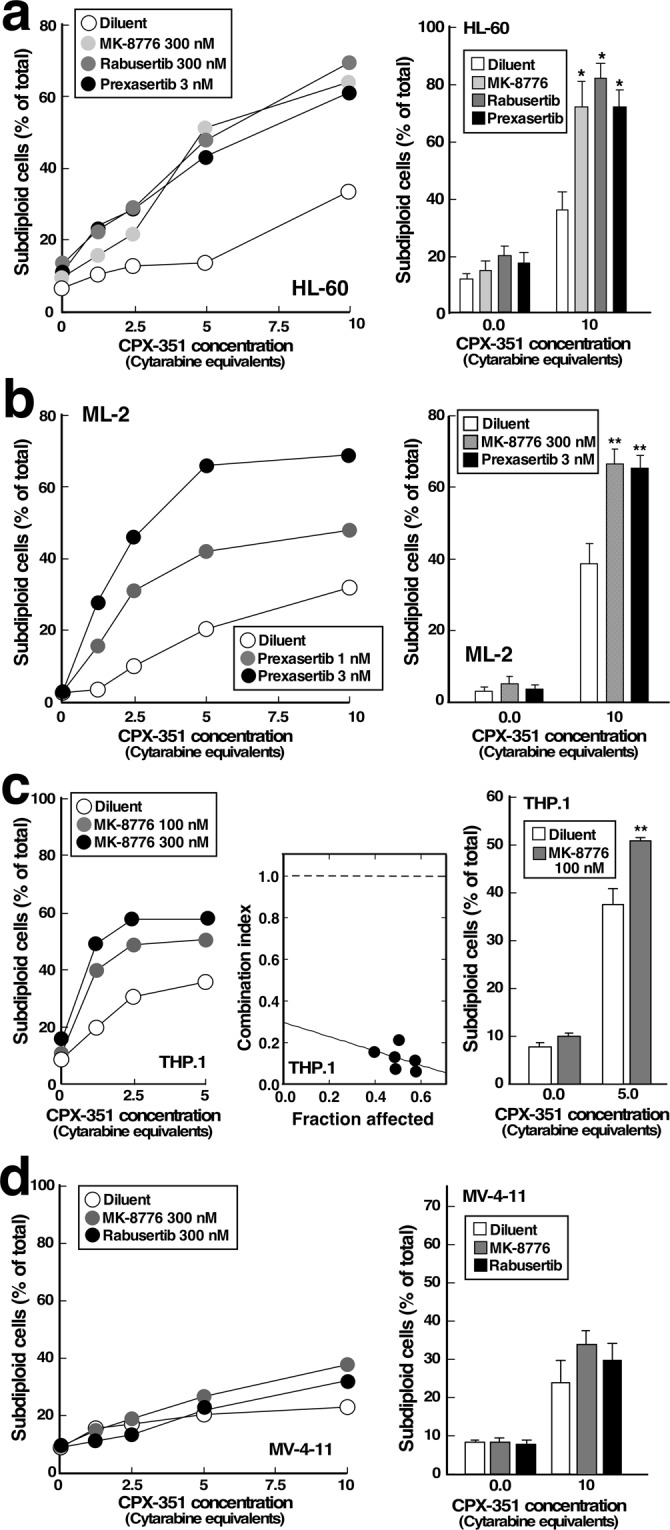
Effect of CHK1 inhibitors on CPX-351-induced apoptosis in additional AML cell lines. HL-60 (**a**), ML-2 (**b**), THP.1 (**c**) or MV-4-11 cells (**d**) were treated for 24 h with varying concentrations of CPX-351 in the absence of presence of MK-8776, rabusertib or prexasertib as indicated, stained with PI and subjected to flow microfluorimetry. Left panels show results from single experiment. Right panels show summary of 3–6 experiments. Results of single-agent MK-8776 in ML-2 cells are shown in Supplementary Figure S4a. Middle panel in c, plot showing combination index values for experiment shown in left panel. A combination index <1 indicates synergy^47^. * and **p < 0.002 (n = 4) and p < 0.02 (n = 3) relative to samples treated with CPX-351 plus diluent.

CHK1 inhibition also increased CPX-351-induced apoptosis in additional AML lines, including HL-60 (Fig. 4a), ML-2 (Fig. 4b and Supplementary Fig. S4a), THP.1 (Fig. 4c), and ML-1 cells (Supplementary Fig. S4b). In particular, MK-8776 sensitized all of these lines to CPX-351. While not every CHK1 inhibitor was tested in every cell line, sensitization was also observed when CPX-351 was combined with rabusertib (Fig. 4a) or prexasertib (Fig. 4a,b). We did not observe any instance where an AML cell line was sensitized to CPX-351 by MK-8776 but not by other inhibitors. Collectively, these observations suggest that CHK1-mediated resistance to apoptosis is not unique to U937 cells. In contrast, MV-4-11 cells were not appreciably sensitized by CHK1 inhibition (Fig. 4d), consistent with the possibility that these *FLT3* mutation-positive cells are resistant to killing as a consequence of some other biochemical process such as alterations in apoptotic signaling^29,30^.

### Effect of CHK1 inhibition on colony forming ability of leukemic cells

**Figure 5:**
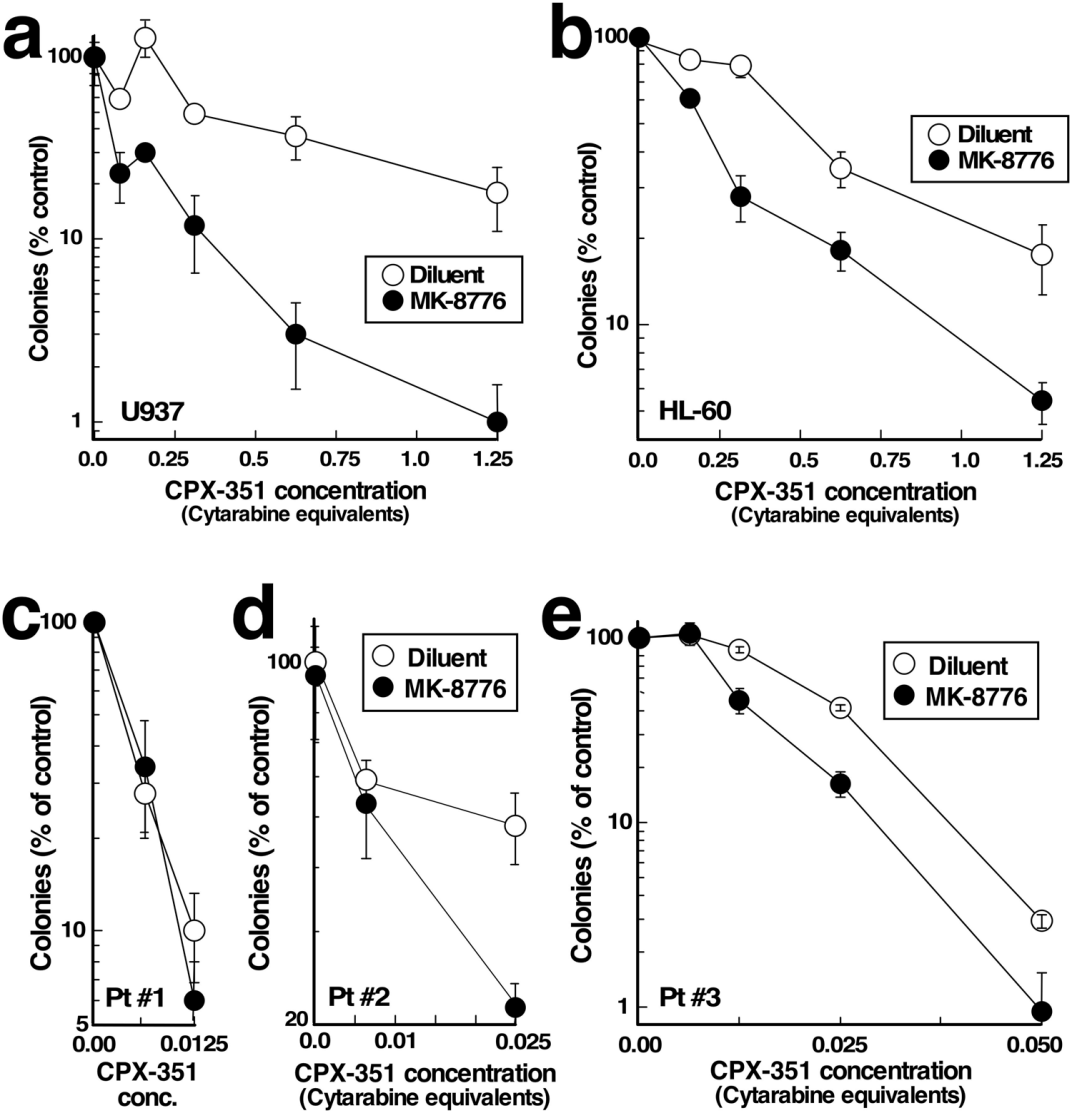
Effects of CPX-351 and MK-8776 on colony formation assays in human AML cell lines and primary AML specimens. (**a**,**b**) U937 (**a**) or HL-60 cells (**b**) were treated for 24 h with CPX-351 alone and in combination with 600 nM MK-8776, washed, plated in soft agar for 12 days and counted. (**c**–**e**) Marrow mononuclear cells from AML patients (Supplementary Table S1) were plated in cytokine-containing Methocult® methylcellulose containing the indicated concentration of CPX-351 in addition to diluent (0.1% DMSO) or 100 nM MK-8776. After a 14-day incubation, leukemic colonies were counted.

Additional assays examined the impact of MK-8776 on the antiproliferative effects of CPX-351 using colony-forming assays in soft agar. In these assays, MK-8776 sensitized U937 and HL-60 cell lines to CPX-351 (Fig. 5a,b). Moreover, in primary AML specimens (Supplementary Table S1), MK-8776 sensitized some AML specimens but not others to CPX-351 (Fig. 5c–e). In particular, sensitization occurred in samples that were relatively resistant to CPX-351 (e.g., Fig. 5e, IC_90_~0.0375 µM cytarabine equivalents) but not in cells that were highly sensitive to CPX-351 (e.g., Fig. 5c, IC_90_~0.01 µM cytarabine equivalents). Because CPX-351 suppresses normal hematopoiesis^16,17^, we examined the impact of the combination on normal marrow colony formation as well. As indicated in Supplementary Fig. S5, MK-8776 also sensitized committed normal progenitors to CPX-351, although their sensitivity did not approach that of sensitive AML samples treated with the combination.

## Discussion

Results of the present study demonstrate that (1) CPX-351 activates the ATR/CHK1-mediated replication checkpoint, (2) CHK1 signaling contributes to CPX-351 resistance, and (3) small molecule checkpoint kinase inhibitors sensitize AML cell lines and clinical samples to CPX-351 *in vitro*. These observations complement and extend our earlier studies indicating that CHK1 inhibition enhances the cytotoxicity of cytarabine as a single agent.

Several observations indicate that CPX-351 activates cell cycle checkpoints. First, cells accumulate in S phase after CPX-351 treatment (Fig. 1a,b). Second, increased phosphorylation of CHK1 on Ser^345^, which reflects ATR activation^23^, is observed within 4.5 hours (Fig. 1c). Third, increased phosphorylation of CHK1 substrates, including KAP1 Ser^473^ and CHK1 Ser^296^, is observed at the same time point, as are decreased CDC25A levels (Figs 1c and S1). All of these changes are consistent with activation of the replication checkpoint^31,32^. In addition, the number of cells traversing mitosis, as manifested by staining for phospho-Ser^10^-Histone H3, is also diminished by CPX-351 (Fig. 1d), suggesting activation of the G2/M checkpoint^24^. These cell cycle perturbations occurred at CPX-351 concentrations below those required to induce apoptosis in a substantial fraction of the cells (cf., Figs 1b and 2b), consistent with earlier results showing that cytarabine as a single agent induces replication checkpoint activation at 10–30 nM but requires 1000 nM to induce apoptosis in AML cell lines over the same 24-hour timeframe *in vitro*^7^.

The CPX-351-induced cell cycle perturbations shown in Fig. 1b,e were examined in the presence of the caspase inhibitor Q-VD-OPh to prevent potential apoptosis. While we are aware that Q-VD-OPh treatment has been associated with increased CDKN1A levels in doxorubicin-treated cells^33^, the work of Rebbaa utilized anthracycline levels 10- to 30-fold higher than those at which cell cycle perturbations were observed in the present study. Moreover, the changes in ATR/CHK1 signaling shown in Figs. 1c and S1 were observed at 4–8 hours in the absence of Q-VD-OPh, arguing that the cell cycle alterations reflect treatment with CPX-351 rather than Q-VD-OPh. Consistent with this interpretation, we were also able to reverse the G2/M checkpoint using a CHK1 inhibitor (Fig. 1d,e).

In further experiments, CPX-351 was used at concentrations achieved at the FDA-approved dose. While some apoptosis was observed with CPX-351 alone, CHK1 knockdown or inhibition increased the apoptosis observed with these higher CPX-351 concentrations in multiple AML cell lines (Figs 2–4). In these experiments, the spectrum of cell lines sensitized by the CHK1 inhibitors was particularly interesting. Among the genomic subsets of AML, those with *TP53* mutations have historically exhibited particularly poor clinical outcomes with cytarabine/anthracycline-based induction therapy^3–5^. Additional studies have suggested that interruption of the replication checkpoint in conjunction with replication stress might be most toxic in cells lacking a G1 checkpoint as a consequence of *TP53* loss or mutation^34–37^. In the present study, we have observed enhanced apoptosis when CHK1 inhibitors are combined with CPX-351 in *TP53* mutant (THP.1) and *TP5*3 null (U937, HL-60) AML lines *in vitro*, consistent with these earlier results. On the other hand, the sensitizing effects of CHK1 inhibitors are not limited to *TP53*-deficient AML cell lines. Instead, sensitization is also observed in *TP53*-wildtype lines such as ML-2 (Figs 4b and S4a) and ML-1 (Supplementary Fig. S4b), as would be expected based on current understanding of the role of the replication checkpoint in stabilization of stalled replication forks^31,32^. Importantly, the sensitizing effects of CHK1 knockdown (Fig. 3a) or CHK1 inhibition (Figs 2, 3 and 4a–c) were often observed at drug concentrations that did not appreciably kill AML cell lines on their own. Small but detectable MK-8776-induced apoptosis was observed in THP.1 cells, permitting analysis of synergy by the median effect method (Fig. 4c). While it was not possible to calculate synergy parameters such as the combination indices in other cell lines such as U937, where CHK1 knockdown or inhibition caused no toxicity, the ability of CHK1 knockdown or inhibition to enhance the effects of CPX-351 without inducing toxicity also meets the definition of synergy^38^.

As indicated above, the CPX-351 concentrations chosen for this study were based on the levels of CPX-351 achieved during treatment of AML patients with this agent^15^. In contrast, we chose CHK1 inhibitor concentrations based on the amount of drug required to inhibit CHK1 autophosphorylation and/or enhance CPX-351 activity. Table 1 compares the concentrations that sensitized to CPX-351 in the present study with drug levels sustained in the clinical setting. Our earlier studies, along with those of others, have demonstrated that MK-8776 enhances the cytotoxicity of cytarabine in AML cell lines and clinical samples *ex vivo*^9,39^. Importantly, the previously observed sensitization was most evident when cells were exposed to cytarabine for 24 h along with 300–600 nM MK-8776, with slightly higher concentrations required to sensitize in colony forming assays than in assays with apoptosis as an endpoint^9^. In the present study, sensitization to CPX-351 was also more evident at MK-8776 concentrations of 300 nM and above. In contrast, mean plasma concentrations at 24 h after administration of MK-8776 as a single agent at its maximum tolerated dose were 80 nM (Table 1), which is below the concentration that effectively sensitizes most AML lines. Dose-limiting off-target effects of MK-8776 on the cardiac conduction system precluded further dose escalation in the clinical setting. As a result, sustained exposure to free MK-8776 levels that would be required to sensitize cells to cytarabine or CPX-351 (Table 1)^40^ cannot readily be achieved clinically.

**Table 1:** CHK1 inhibitor concentrations required to sensitize AML lines to CPX-351.

Inhibitor	Concentration to sensitize to CPX-351 *in vitro*	Mean plasma concentration 24 h after administration at MTD*	Reference
MK-8776	300–600 nM (Figs 2 and 5a)	30 ng/ml = 80 nM	^40, 48^
Rabusertib	300 nM (Figs 3b and S4a)	1000 ng/ml = 2.5 µM	^49^
Prexasertib	3 nM (Figs 3c and 4b)	15 ng/ml = 41 nM	^42^

*Indicates the mean plasma concentration 24 h after the administration of the indicated inhibitor at the maximum tolerated dose (MTD) as a single agent except in the case of MK-8776, where the MTD with cytarabine is similar to that as a single agent as indicated in the cited references.

On the other hand, we also observed that the recently described CHK1 inhibitor prexasertib^28^ enhances the cytotoxicity of CPX-351 at low nanomolar concentrations (Figs 3e, 4a,b). Unlike MK-8776, prexasertib lacks detectable cardiac toxicity in the clinical setting at the maximum tolerated dose^41^. Moreover, prexasertib exposures in the clinical setting^42^ far exceed those required to sensitize to CPX-351 (Table 1), raising the possibility that further study of CPX-351 in conjunction with prexasertib might be particularly fruitful.

## Methods

### Materials

Reagents were purchased as follows: MK-8776 and rabusertib (LY2603618) from Chemietek (Indianapolis, IN); cytarabine, daunorubicin and propidium iodide (PI) from Sigma-Aldrich (St. Louis, MO); prexasertib (LY2606368) from Selleckchem (Houston, TX); Q-VD-OPh from SM Biochemicals (Anaheim, CA); murine monoclonal anti-CHK1 (catalog #sc-8408) from Santa Cruz Biotechnology (Santa Cruz, CA); murine monoclonal anti-phospho-Ser^473^-KAP1 from Biolegend (San Diego, CA); murine monoclonal anti-phospho-Ser^139^-histone H2AX (catalog #05-636) and rabbit polyclonal anti-phospho-Ser^10^-Histone H3 from Millipore (Billerica, MA); rabbit polyclonal antibodies to CDC25A (catalog #ab989) and KAP1 (catalog #ab10484) as well as rabbit monoclonal antibodies to CHK2 and phospho-Thr^68^-CHK2 (catalog #ab109413 and ab32148) from Abcam (Cambridge, MA); anti-caspase 3 (catalog #610323) from BD Biosciences (San Jose, CA); and rabbit polyclonal antibodies to phospho-Ser^296^-CHK1 (catalog #2349) and phospho-Ser^345^-CHK1 (catalog #2341) as well as rabbit monoclonal antibodies to glyceraldehyde phosphate dehydrogenase (catalog #2118), cleaved caspase 3 (#9664), PARP1 (catalog #9532), and total histone H2AX (catalog #7631) from Cell Signaling Technology (Beverly, MA). Murine monoclonal antibody H90-10 to heat shock protein 90β (HSP90β) was a kind gift from David Toft (Mayo Clinic, Rochester, MN). CPX-351 was provided by Lawrence Mayer (Celator Pharmaceuticals, Princeton, NJ) prior to the merger of Celator with Jazz Pharmaceuticals.

### Cell culture

U937 and THP.1 cells (American Type Culture Collection, Manassas, VA) as well as HL-60 (Robert Abraham, Pfizer, La Jolla, CA), ML-1 (Michael Kastan, Duke University, Durham, NC), ML-2 (James Bogenberger, Mayo Clinic, Scottsdale, AZ) and MV-4-11 cells (Terra Lasho, Mayo Clinic, Rochester, MN) were authenticated by short tandem repeat profiling, reinitiated from frozen stocks at 3-month intervals, and maintained in RPMI 1640 containing 10% FBS, 100 U/ml penicillin G, 100 μg/ml streptomycin, and 2 mM glutamine (medium A) at concentrations below 1 × 10^6^ cells/ml at all times. The *TP53* mutation status of each line is as follows: *TP53* null: HL-60, U937; *TP53* mutant: THP.1 (pR174fs); *TP53* wildtype: ML-1, ML-2 and MV-4-11. Aliquots were diluted to 2–4 × 10^5^ cells/ml in medium A and treated with CPX-351 (added from 10X stocks freshly prepared in medium A) and CHK1 inhibitors (added from 1000X stocks in DMSO) for specific assays below.

Small interfering RNAs (siRNAs) were transiently transfected into U937 cells by electroporation (280 V, 10 ms) using a BTX 830 square wave electroporator (BTX, San Diego, CA) under conditions described previously^43^. siRNAs utilized included non-targeting control siRNA (ThermoFisher, Foster City, CA; catalog #AMB4635,), siCHK1 #1 (5′-GGAGAG-AAGGCAAUAUCCAtt-3′ (Thermo Fisher catalog #106), and siCHK1 #2 5′AAGCGU-GCCGUAGACUGUCCAtt3′(Dharmacon, Lafayette, CO, cat# HACJA-000033).

### Analysis of cell cycle distribution and apoptosis

After incubation^7^ for 24 h with CPX-351 in the absence or presence of CHK1 inhibitors, cells were resuspended in ice cold 0.1% (wt/vol) sodium citrate containing 50 µg/mL PI and 0.1% (wt/vol) Triton X-100, incubated at 4 °C overnight, and analyzed by flow microfluorimetry in the FL2 channel on a Becton Dickinson (Franklin Lakes, NJ) FASCanto II flow cytometer. After collection of 20,000 events, files were analyzed using Modfit (Verity Software, Topsham, ME) for cell cycle distribution or Becton Dickinson CellQuest software for events with <2n DNA content. Alternatively, cells were fixed in 3:1 methanol:acetic acid, dropped onto coverslips and stained with 1 µg/ml Hoechst 33258 in 50% glycerol containing PBS, and examined for apoptotic morphological changes as previously described^6,7^.

To assess the G2/M checkpoint, cells were treated for 24 h as indicated in individual figures, fixed in 70% (v/v) ethanol, rehydrated in PBS, and simultaneously stained with antibody to phospho-Ser^10^-Histone H3 and propidium iodide as initially described by Xu *et al*.^24^.

### Immunoblotting

U937 cells were exposed to the indicated CPX-351 concentrations without or with MK-8776 for 4–8 hours as indicated, washed with PBS, and lysed in 2X SDS-PAGE sample buffer (1 × 10^7^ cells/mL). Lysates (2 × 10^5^ cells/lane) were separated by SDS-PAGE, transferred to Immobilon P, and blotted for the indicated antigens. Alternatively, cells treated with siRNAs or with drugs for 24 hours were lysed in 6 M guanidine hydrochloride under reducing conditions and prepared for SDS-PAGE as described^44^.

### Additional assays

Assays for the ability of AML cell lines to form colonies in 0.3% agar were performed as described^7,9^.

### Leukemia and normal samples

After informed consent was obtained under the aegis of protocols approved by the Mayo Clinic Institutional Review Board and Johns Hopkins University Institutional Review Board, bone marrow aspirates were acquired from AML patients in accordance with relevant guidelines and regulations prior to therapy. Mononuclear cells were isolated on ficoll-Hypaque gradients, washed with ice cold serum-free RPMI 1640 medium, resuspended at 1.5 × 10^6^ cells/ml in Iscove’s modified Dulbecco’s medium containing 20% (vol/vol) heat-inactivated fetal bovine serum (FBS), 100 U/ml penicillin G, 100 μg/ml streptomycin, and 2 mM glutamine, and plated at 300,000 cells/plate in MethoCult® methylcellulose (StemCell Technologies, Vancouver, British Columbia) containing the indicated concentrations of CPX-351 and MK-8776. Leukemic colonies were counted after a 14-day incubation^45^. WHO classification and karyotype were determined during the routine clinical care of the AML patients^46^.

Anonymized freshly isolated normal marrow samples, obtained from hip arthroplasty specimens, were provided by the Henry J. Predolin Biobank for Hematology Research (Mayo Clinic, Rochester, MN). After cells were cultured in Methocult® for 14 days as described above, normal myeloid colonies were scored^45^. Results are presented as the sum of BFU-E, CFU-GM and CFU-M, all of which showed similar sensitivities.

### Statistical analysis

Experiments were performed 3–10 times independently as indicated in the figure legends. Statistical analyses were performed using StatView v.5 (SAS Institute). Results of various treatments were compared using 2-tailed *t* tests, with p < 0.05 considered to be statistically significant. Where multiple treatments were compared simultaneously, we conducted analysis of variance with post hoc *t* tests and applied a Bonferroni correction for multiple comparisons to all p values. Results of multiple experiments are summarized as bar graphs showing mean ± sd. To assess the presence of synergy, combination indices were calculated by the method of Chou and Talalay^47^ under the assumption that effects were mutually exclusive, which is equivalent to isobologram analysis^38^. With this method, synergy is indicated by a combination index <1, additivity by a combination index equal to 1, and antagonism by a combination index >1^47^.

## Supplementary information

Supplementary information for publication
