# Supplementary material for: Effect of CHK1 Inhibition on CPX-351 Cytotoxicity in vitro and ex vivo
Source: Sci Rep. 2019 Mar 5;9:3617. doi: 10.1038/s41598-019-40218-0 (PMC6400938; doi:10.1038/s41598-019-40218-0)
Supplement: Supplementary file 1 — Supplementary information for publication [file 41598_2019_40218_MOESM1_ESM.pdf]

Supplementary Material for

# Effect of CHK1 Inhibition on CPX-351 Cytotoxicity *in vitro* and *ex vivo*

Nicole D. Vincelette, Husheng Ding, Amelia M. Huehls, Karen S. Flatten, Rebecca L. Kelly, Mira A. Kohorst, Jonathan Webster, Allan D. Hess, Keith W. Pratz, Larry M. Karnitz, and Scott H. Kaufmann

**Table S1**
**Description of Patients Providing Samples for Figure 5**

| Pt # | Sex | Age (y) | WHO | Karyotype | Prior hemato-logical disorder | FLT3 mutation | Newly diagnosed or relapsed |
|---|---|---|---|---|---|---|---|
| 1 | M | 60 | AML, NOS | 46 XY, inv(9) | MDS | - | Relapsed |
| 2 | F | 59 | AML, NOS | 47 XX, inv(16) +22 | - | - | Newly diagnosed |
| 3 | F | 63 | AML with monocytic differentiation | 46 XX | - | - | Relapsed after prior BMT |

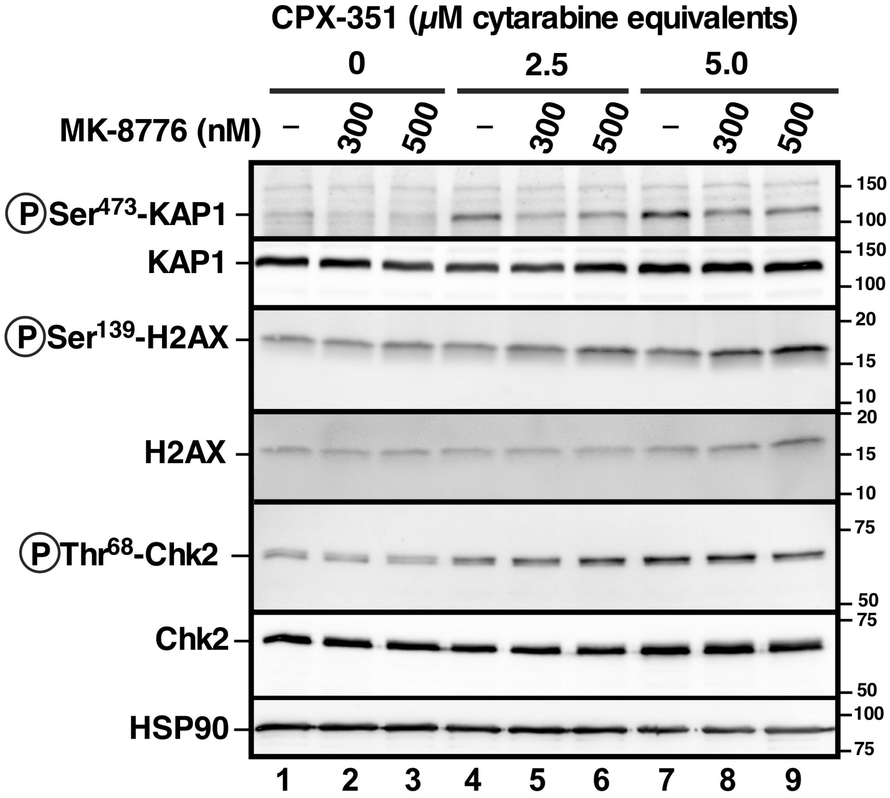


**Supplementary Figure S1. Effects of CPX-351 and MK-8776 on phosphorylation of additional DNA damage response proteins.** U937 cells were treated for 8 hours with CPX-351 (2.5 or 5 μM cytarabine equivalents) and diluent or the indicated concentration of MK-8776. Cell lysates were then subjected to SDS-PAGE and immunoblotting for the indicated antigens.

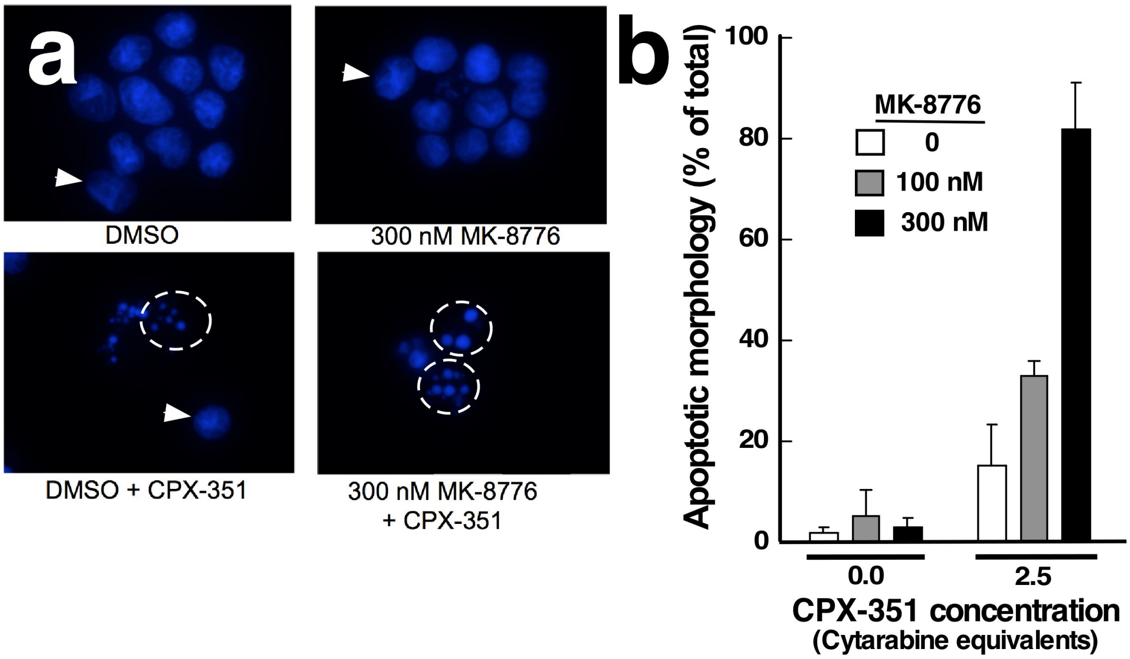


**Supplementary Figure S2. CPX-351 in combination with MK-8776 increases apoptotic morphology.** U937 cells were treated for 24 h with CPX-351 ± 100 or 300 nM MK-8776, harvested, fixed and stained with Hoechst 33358. 300 cells were counted per treatment. **a,** nuclear morphology of untreated cells, and cells treated with CPX-351 alone or in combination with MK-8776. Arrowheads, examples of normal appearing nuclei. Dashed circles, examples of nuclear fragments in single apoptotic cells. Each cluster of apoptotic fragments was counted as one apoptotic nucleus. **b,** quantification of samples from experiments shown in panel a and additional samples.

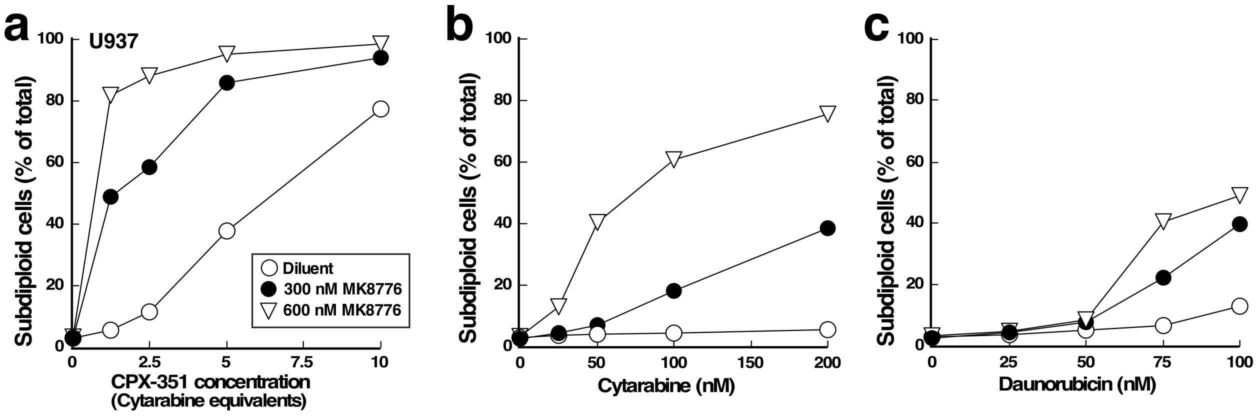


**Supplementary Figure S3. Effect of MK-8776 on apoptosis induction in U937 cells by CPX-351, cytarabine or daunorubicin.** U937 cells were treated for 24 h with the indicated concentrations of CPX-351 (**a**), cytarabine (**b**), or daunorubicin (**c**) ± 300 or 600 nM MK-8776. At the conclusion of the incubation, cells were stained with PI and analyzed by flow microfluorimetry to detect subdiploid events.

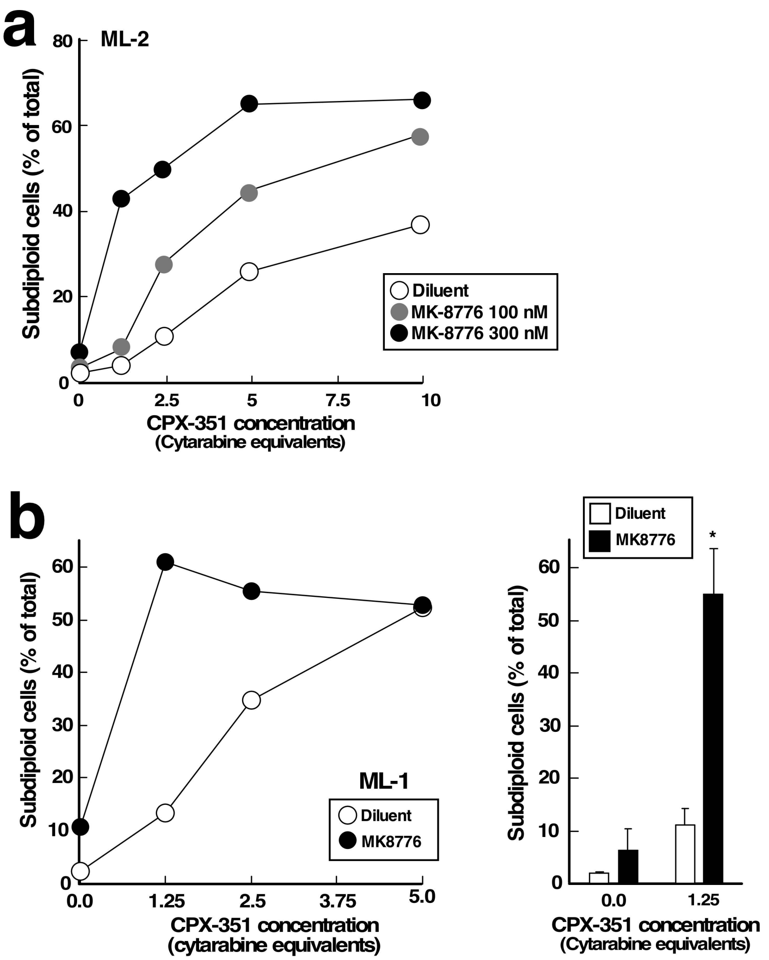


**Supplementary Figure S4. CHK1 inhibitors enhance CPX-351-induced apoptosis in TP53-wildtype ML-2 and ML-1 cells.** **a,** ML-2 cells were treated for 24 h with CPX-351 in the absence or presence of MK-8776, stained with PI and subjected to flow microfluorimetry. Shown is one of three independent experiments. Summarized results from these experiments are shown in the right panel of Fig. 4b of the main text. **b,** ML-1 cells were treated for 24 h with CPX-351 in the absence and presence of MK-8776, stained with PI and subjected to flow microfluorimetry. **Left panel,** results from one of three experiments. **Right panel,** summary of 3 independent experiments under conditions shown in left panel. Error bar, ± SD. *, $p = 0.02$ relative to CPX-351 alone by unpaired *t* test.

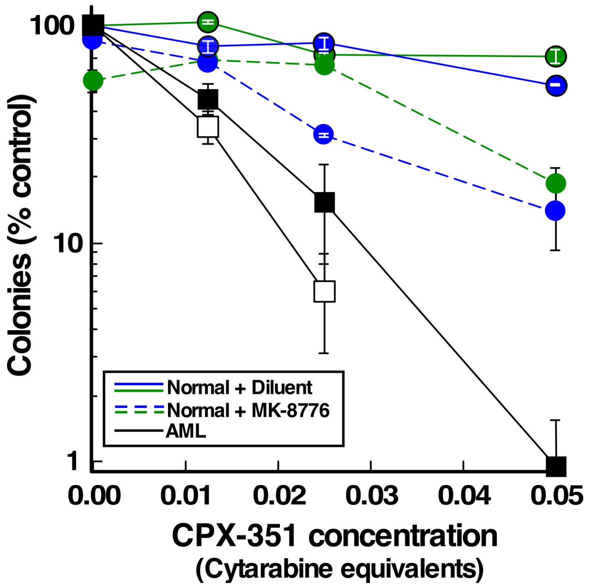


**Supplementary Figure S5. Effect of MK-8776 on colony formation by normal myeloid cells.** Marrow mononuclear cells from two normal subjects (blue and green lines, respectively) were plated in Methocult® in the indicated concentration of CPX-351 and diluent (solid lines) or 100 nM MK-8776 (dashed lines). After 14 days, normal erythroid and myeloid colonies were counted and normalized to the number of colonies that formed in the absence of drug treatment. Shown is the sum of BFU-E, CFU-GM and CFU-M, each of which showed a similar pattern of sensitivity, relative to samples treated with diluent but no drug. For comparison, results observed with the two sensitive AML samples in the presence of 100 nM MK-8776 (Fig. 5c and 5d) are also shown (black lines).

# Figure 1

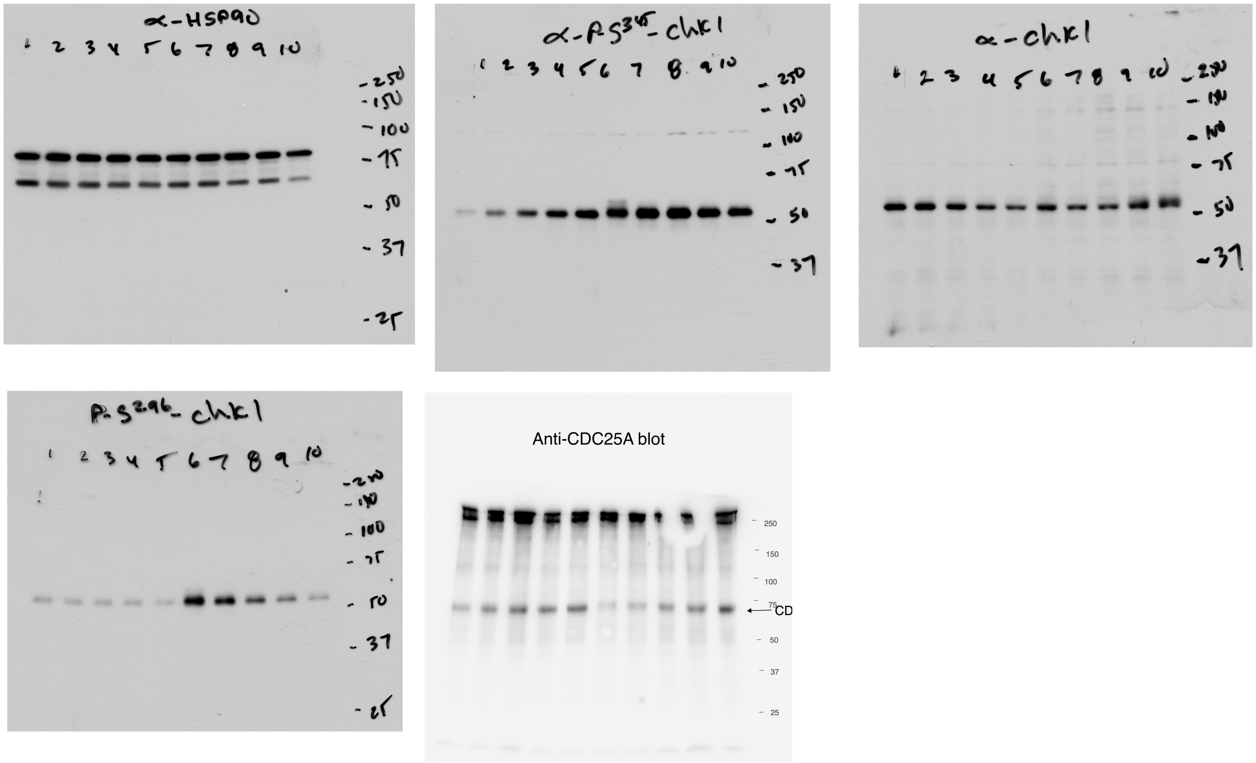
α-HSP90
α-P-S345-chk1
α-chk1
P-S296-chk1
Anti-CDC25A blot
250
150
100
75
50
37
25
CD

**Figures 2 and 3**

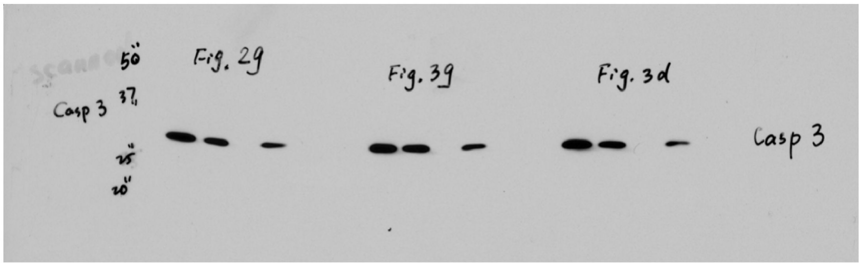


Caspase 3

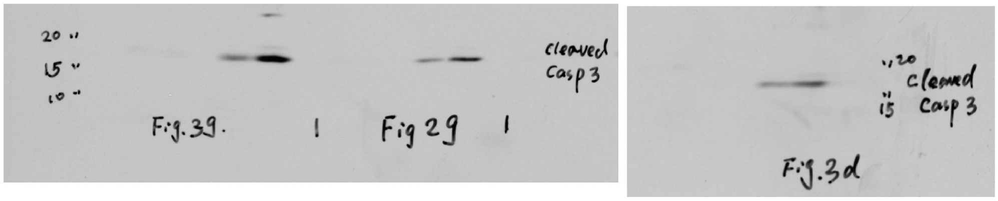


Cleaved caspase 3

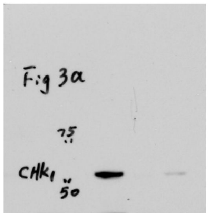


CHK1

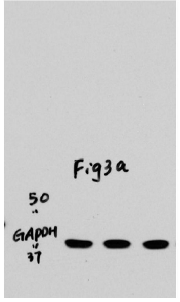


GAPDH

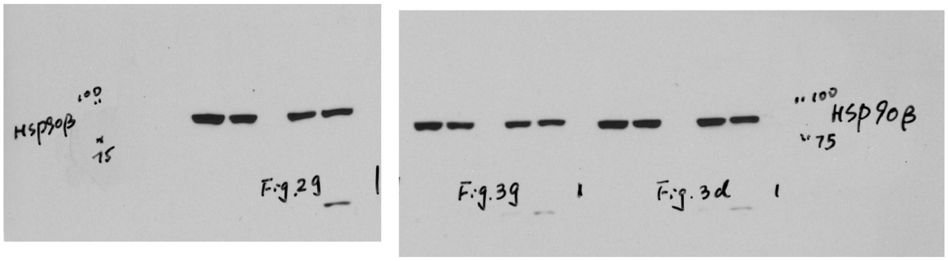


HSP90

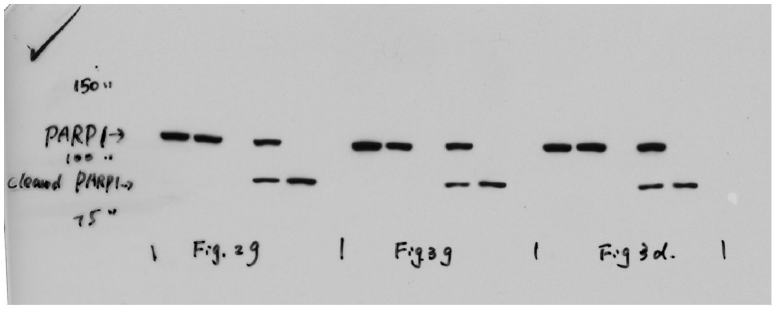


PARP1

# Figure S1

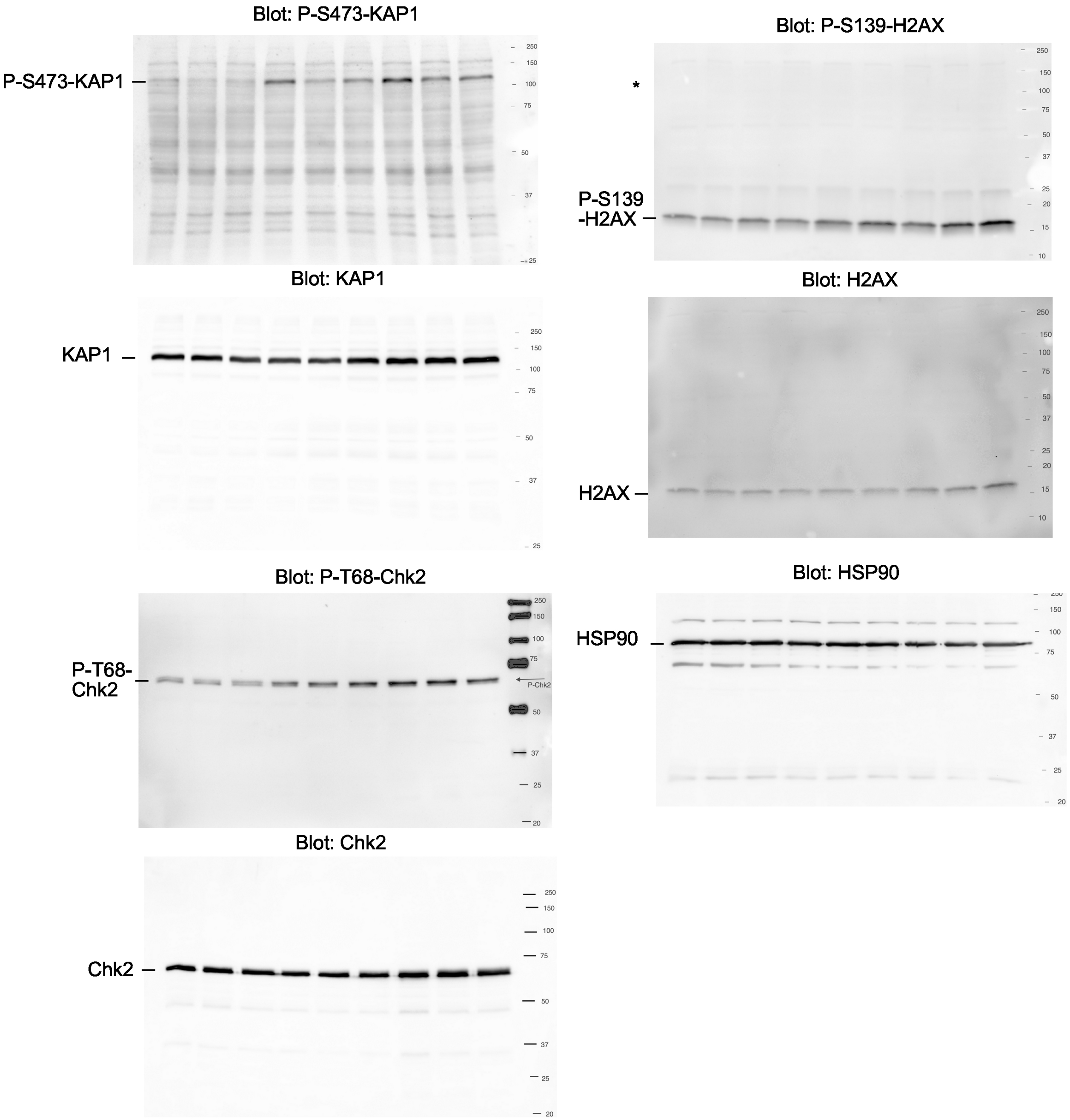


**Figure S6. Original scans of blots shown in various figures as indicated.**
